# An Update on Spinal Dural Arteriovenous Fistulae: Case Series and Systematic Review

**DOI:** 10.7759/cureus.65537

**Published:** 2024-07-27

**Authors:** George Vavoulis, Dimitrios Giakoumettis, Abraham Tsitlakidis, Aikaterini Karagianni, Bilal Almasarwah, Theodoros Vogiatzoglou, Evropi Amanatidou, Konstantinos Rados, Konstantinos Vlachos

**Affiliations:** 1 Neurosurgery, KAT Attica General Hospital, Athens, GRC; 2 Neurosurgery, Agios Savvas Attica General Hospital, Athens, GRC; 3 Neurological Surgery, KAT Attica General Hospital, Athens, GRC

**Keywords:** embolization, surgery, myelopathy, spinal dural fistulae, spinal vascular shunts, systematic review

## Abstract

Spinal dural arteriovenous fistulae (sDAVFs) are rare entities with delayed diagnosis, potentially dramatic clinical manifestations, and complex management. We aim to present our mini case series and perform an updated systematic review of the usual patient profile, to search for established prognostic factors, to compare the effectiveness and safety of surgical and endovascular intervention, and to discuss trends in therapeutic strategy. We retrospectively collected data from patients treated in our department in the last decade (2014-2024) and we systematically reviewed the literature according to the PRISMA (Preferred Reporting Items for Systematic Reviews and Meta-Analyses) criteria and searched the PubMed database for relevant articles published in the same period. Epidemiologic data, imaging modalities, prognostic factors, and treatment modalities were assessed. Firstly, we identified four illustrative cases from our institution. In addition, our search yielded 559 studies and our review included 82 original studies. 3130 patients were identified (mean age 61; male-to-female ratio 3:1). Most commonly, the fistula level was in the thoracic spine (65%). Surgery was provided to 1837 patients (1213 as primary treatment) and embolism to 1085 (932 as primary treatment). Initial fistula occlusion rate and recurrence rate were 98.1% and 1.9% for surgery and 71.1% and 9.6% for embolism, respectively. No difference between the two modalities with respect to clinical outcome was observed. SDAVFs remain a challenge for neurosurgeons concerning both diagnosis and management. Surgery remains superior to embolism with respect to success as an initial treatment. Embolism can be offered if certain contraindications do not coexist. All symptomatic patients should be offered treatment, whereas asymptomatic patients could be conservatively managed only if the close patient follow-up can be secured.

## Introduction

Spinal arteriovenous shunts compose 4-5% of the spinal intradural lesions. The majority of the shunts, about 70%, refer to spinal dural arteriovenous fistulae (sDAVFs). The latter are divided into dorsal and ventral according to Spetzler’s classification [1] and compose the formerly known type I shunts [2]. The fistula is supplied arterially by the radicular artery (RA) or radiculomeningeal branch (RMenA) of the segmental artery and drained in 60% of the cases by the radicular (RV) or radiculomedullary (RMedV) vein and in 40% by a bridging vein that joins the epidural venous plexus (EVP) [3]. sDAVFs differ anatomically from spinal epidural arteriovenous fistulae (sEAVFs), as the latter is a high-flow shunt, supplied by an epidural artery, most commonly the dorsal somatic branch (DSB) of the segmental artery or a distal branch of the spinal artery and drained by the EVP directly and only rarely with RMedV participation [4-6]. Due to this anatomical difference, sDAVFs present with different management strategies [4,6]. Moreover, in the majority of cases, there is a significant time window from symptom onset to definite diagnosis and treatment, during which there is notable disease progression. Therefore, sDAVFs shape a challenging neurosurgical scenario with respect to achieving applicable diagnostic alertness, resorting to more or less aggressive management, and providing evidence-based informed patient consent. Our objective was to delineate the patient profile, look for established prognostic factors, compare treatment modalities' effectiveness and safety, and discuss optimal investigation and therapeutic strategy.

## Case presentation

Case 1

**Figure 1:**
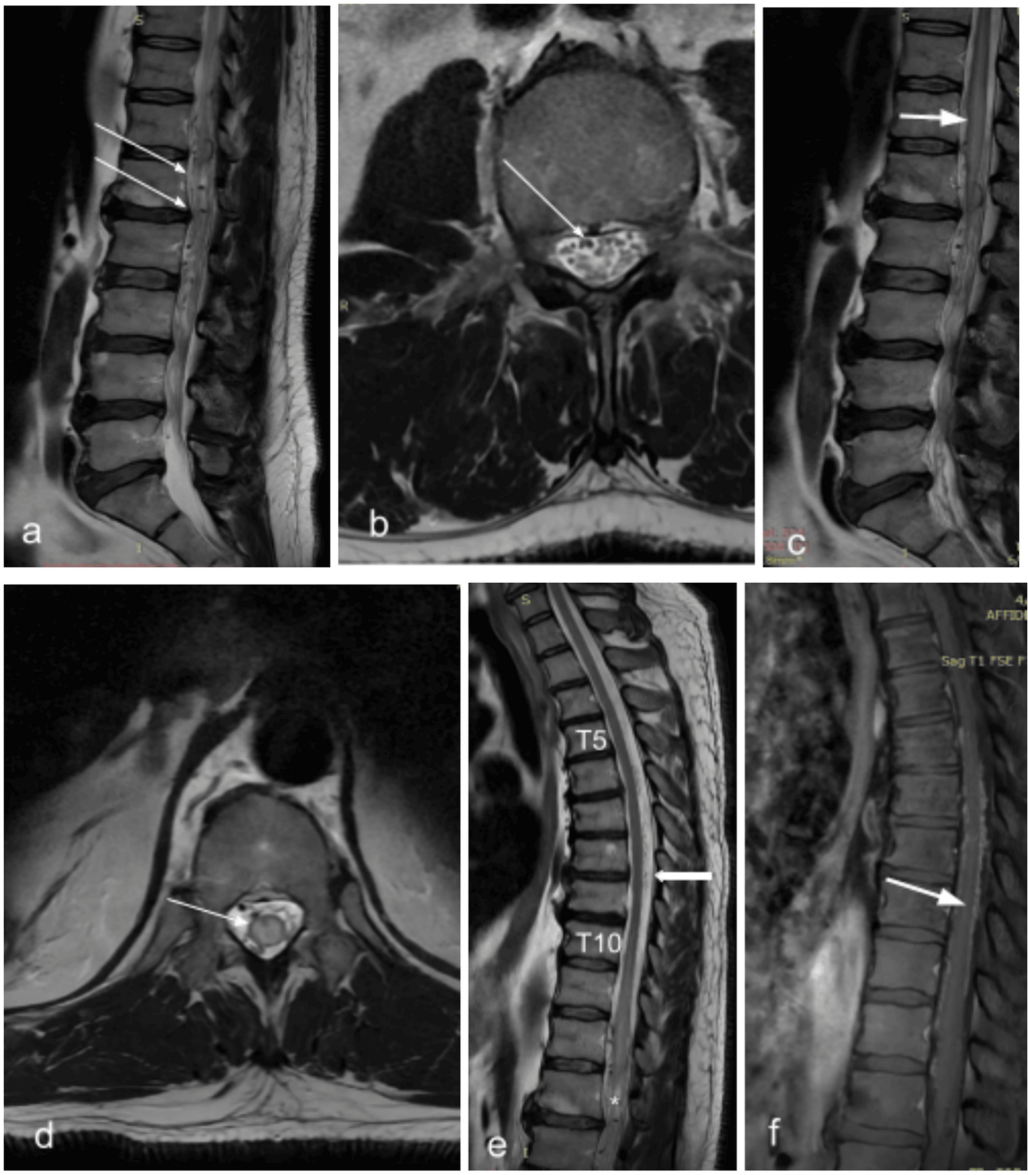
Pre-operative MRI of patient #1. a: Serpentinous intradural extramedullary flow voids in sagittal lumbar T2-weighted image (T2WI) (arrows). b: Descending vein between the rootlets of equina in axial T2WI (arrow). c: Sagittal T2WI from Th10 to L2 level, shows the intramedullary cord oedema (arrow). d: Same in the axial T2WI (arrow). e: Dorsal perimedullary venous plexus ascending as rostrally as levels Th5 to Th10 (arrow). f: post-gadolinium (PG) image discloses the intramedullary enhancement at the same levels as the oedema (arrow).

**Figure 2:**
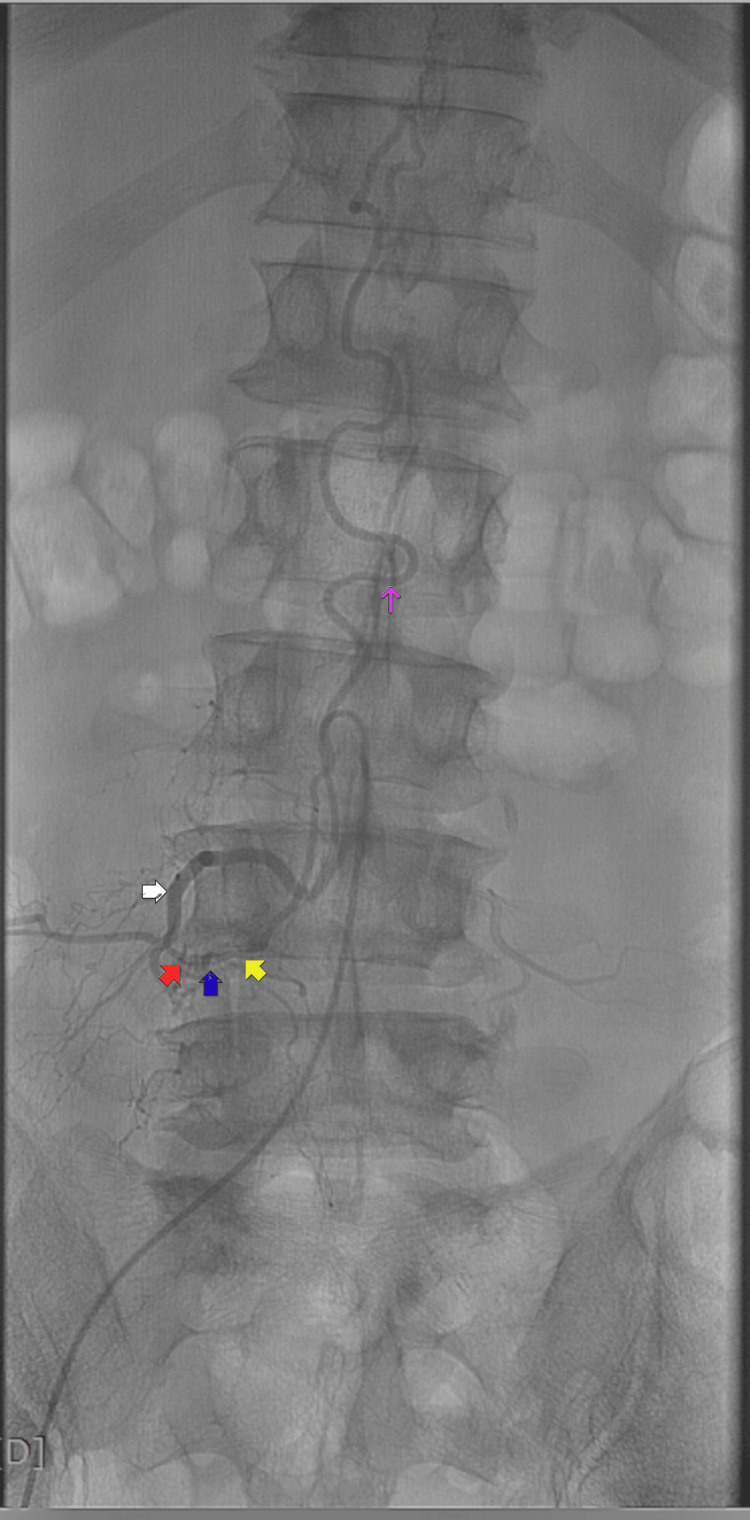
Preoperative digital subtraction angiography (DSA) of patient #1. Anteroposterior (A-P) image, shows the anatomy of the spinal dural arteriovenous fistula (sDAVF): the segmental artery (white arrow) of the right L4 spinal root, starts ventrally in front of and rounding the vertebral body of L4, then passes through the anterior superior quadrant of the foramen (subpedicular), to give off the radicular artery (RA) (red arrow); the fistula (blue arrow) between the RA and radiculomedullary (RMedV) vein (yellow arrow); the congested posterior spinal vein (purple arrow) ascending multiple levels above the fistula level.

**Figure 3:**
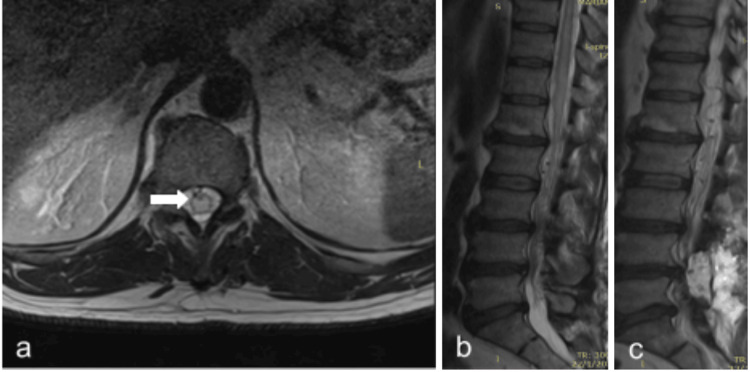
Pre-operative MRI of patient #1. a: Serpentinous intradural extramedullary flow voids in sagittal lumbar T2WI (arrows). b: Descending vein between the rootlets of equina in axial T2WI (arrow). c: Sagittal T2WI from Th10 to L2 level, shows the intramedullary cord oedema (arrow). d: Same in the axial T2WI (arrow). e: Dorsal perimedullary venous plexus ascending as rostrally as levels Th5 to Th10 (arrow). f: post-gadolinium (PG) image discloses the intramedullary enhancement at the same levels as the oedema (arrow).

**Figure 4:**
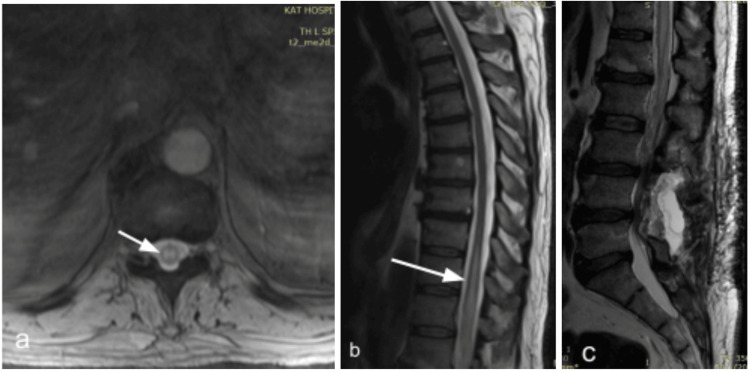
Postoperative MRI (second surgery) of patient #1. a: Persistent cord oedema in axial T2WI (arrow). b: same in sagittal T2WI. c: perimedullary venous plexus has been decongested.

**Figure 5:**
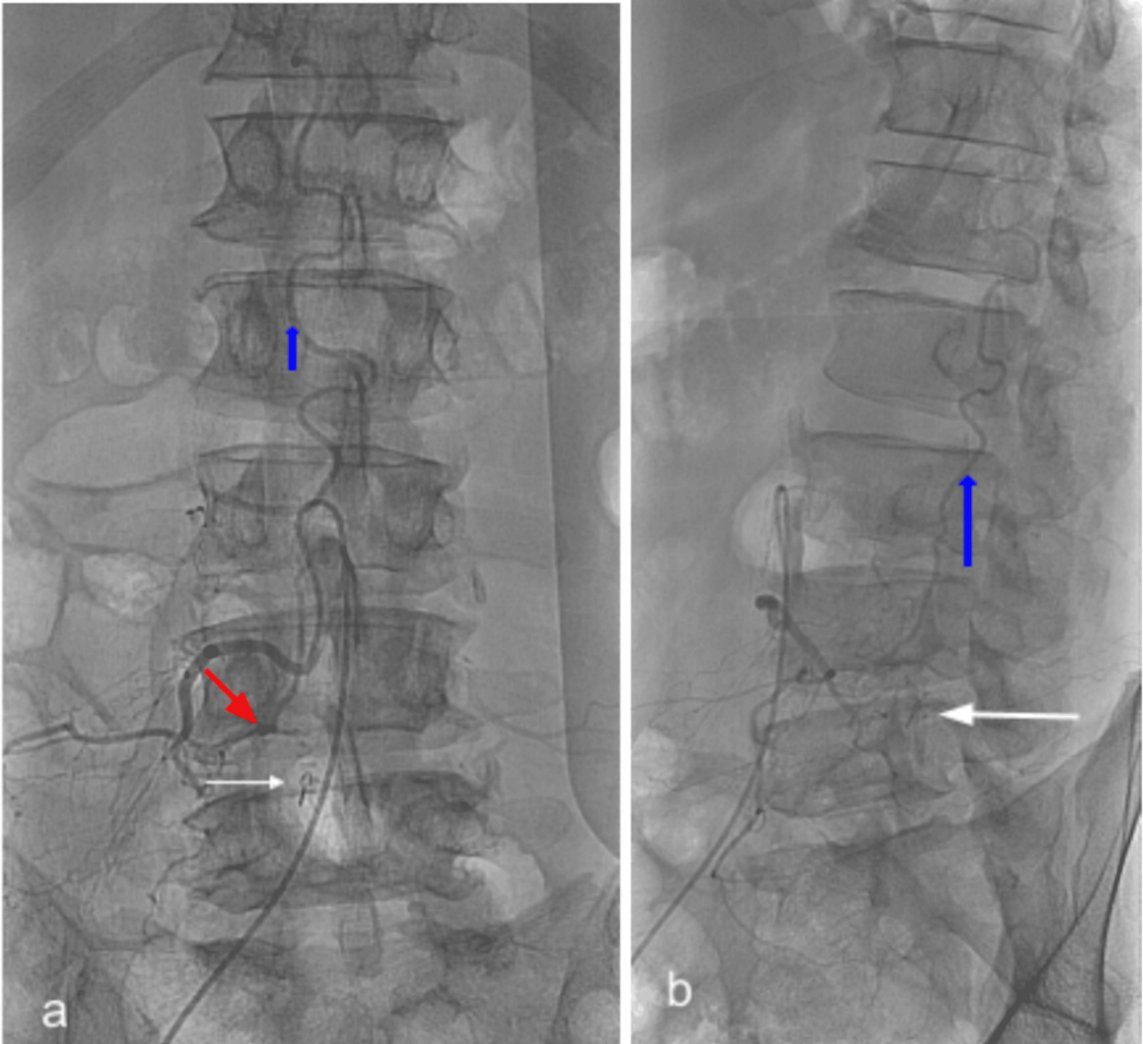
Postoperative (first surgery) digital subtraction angiography (DSA) of patient #1. Anteroposterior (A-P) (a) and lateral (b), demonstrate the extradural dorsally positioned clip (white arrow) at the L4-5, moved from its immediate postoperative position and the residual fistula (red arrow) located intradurally and more laterally, while the congestion of the posterior spinal vein (blue arrow) remains.

**Figure 6:**
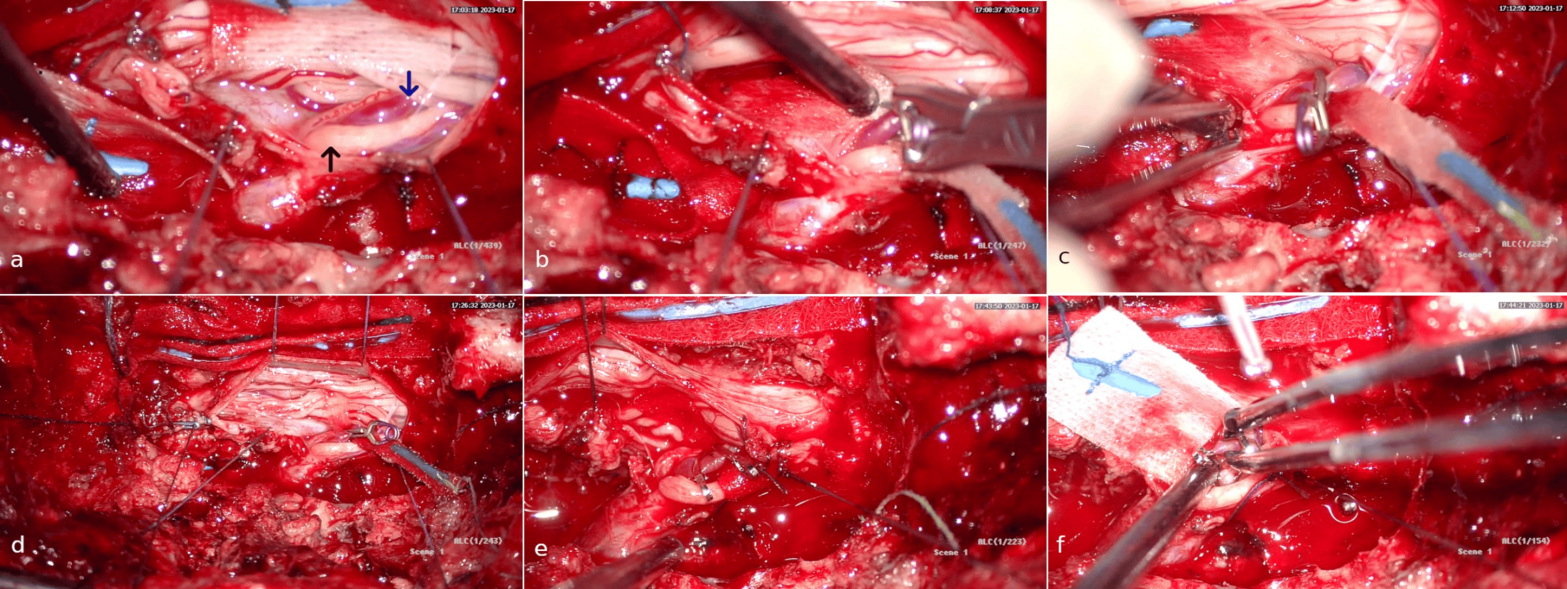
Intraoperative image of patient #1 (second surgery). a: Dura opening and recognition of the root (black arrow) and radicular vein (RV) (blue arrow). b: Temporary clipping of the arterialized vein. c,d: Notice the proximal decongestion of the vessel. e: placement of the second clip. f: fistula is interrupted through cauterization and division of the vessel after revalidating through unchanged somatosensory potentials (SSEPs) that the vessel being clipped is neither the radicular artery (RA) nor the radiculomeningeal artery (RMenA).

**Figure 7:**
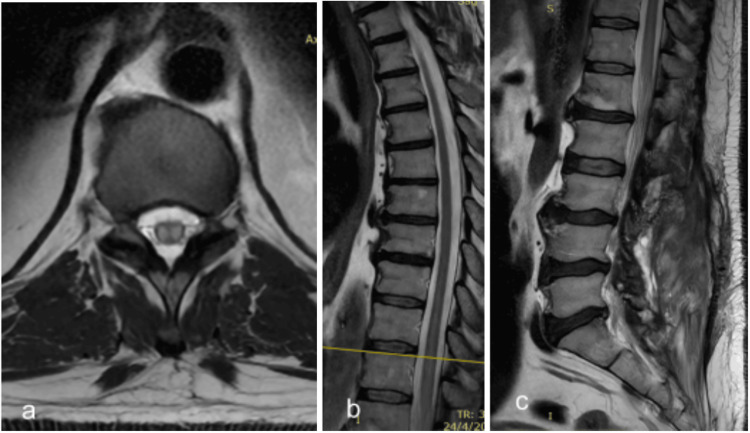
Three-month follow-up MRI of patient #1. a (axial T2WI) and b (sagittal T2WI): Oedema is still persistent in the sagittal T2WI and its respective level (marked with the yellow line in (b)) on the axial T2WI. c: T2WI shows no evident residual serpiginous flow voids.

A 58-year-old male presented with a 3-month lumbar pain radiating to the right leg with numbness, worse with exercise, and unremarkable physical examination. The imaging study included magnetic resonance imaging (MRI), angiography (MRA), and digital subtraction angiography (DSA). On T2 weighted imaging (T2WI) he had hyperintense intramedullary signal extending from Th9 level down to the conus, and perimedullary vascular flow voids dorsally at the lower thoracic and lumbar levels, enhanced on post-gadolinium (PG) T1-weighted image (T1WI) (Figure 1). The DSA (selective catheterization of the internal iliac arteries (IIA), Th6-Τh12 posterior intercostal and lumbar arteries on both sides) revealed a Spetzler type I (intradural of the nerve root sleeve) fistula with the right L4 RMenA, draining into the perimedullary venous plexus that ascended four levels (Figure 2). He underwent L4-5 fenestration to reveal the feeding artery and the draining vein, which was adherent to one of the cauda equina rootlets. A temporary clip was placed extradurally over the draining vein for 10 minutes to evaluate fluctuations in motor-evoked potentials (MEP) and visually assess the decongestion of the draining vein. The draining vein was then cauterized at its proximal - to the fistula - end, and the temporary clip was replaced with a permanent one. The patient underwent postoperative MRI which demonstrated persistent intramedullary oedema and serpiginous venous congestion and venous congestion (Figure 3). Nevertheless, the patient reported improvement of symptoms. His postoperative hospital stay was uneventful, and the patient was discharged ambulatory, only to return 4 days later (14 days postoperatively) with acute onset of cauda equina syndrome, unable to walk. Subsequent MRI reconfirmed persistent cord edema with compression signs (Figure 4) and the DSA showed persistent flow at L4 RV and ascending spinal vein (Figure 5). He underwent L3 and L4 laminectomy, and additionally, the L4 RV was cauterized intradurally only after it was identified to be engorged (Figure 6). He was discharged four days later, ambulatory, with minimal assistance and a strict follow-up plan. At the 3-month follow-up, the patient was ambulatory with only a residual right leg numbness and unremarkable urodynamic testing. His MRI (Figure 7) and DSA at the time showed the perimedullary plexus decongested, without any evident feeder vessel, and medullary oedema in remission.

Case 2

**Figure 8:**
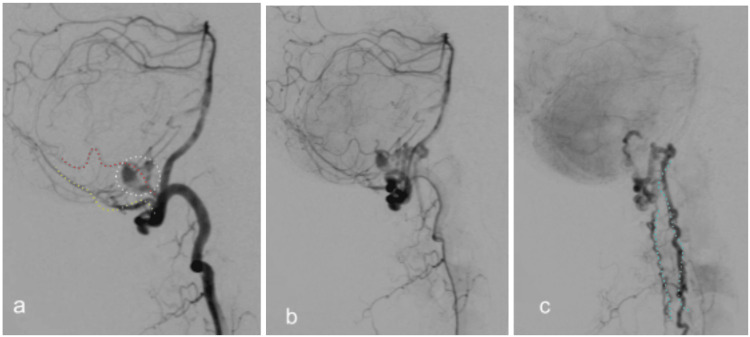
Preoperative digital subtraction angiography (DSA) of patient #2. Early (a,b) and late (c) phase. The fistula, along with the venous aneurysm (white circle), is at the posterior-left wall of the foramen magnum, fed by the posterior meningeal artery (PMA) (yellow dotted line) and small branches of the left posterior spinal artery (PSA) (red dotted line) and drained by the dilated perimedullary veins (cyan dotted lines).

**Figure 9:**
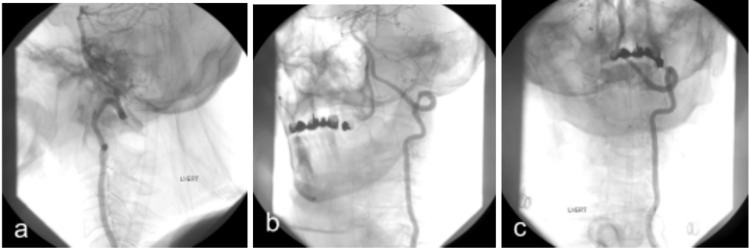
Postoperative DSA of patient #2. Lateral (a), oblique (b), Anteroposterior (A-P) (c). No evidence of residual fistula.

A 70-year-old male patient presented in the emergency department due to sudden-onset tetraparesis. Clinical assessment revealed bilateral spastic tetraplegia (muscle strength 1/5, positive Babinski and Hoffmann's signs) and complete bilateral hypesthesia below the Th3 dermatome level. An emergency cervical and brain MRI showed a myelopathic signal extending from the medulla oblongata to C6-7 due to compression from dorsal dilated intradural vessels at the foramen magnum. The patient quickly developed respiratory distress and was intubated. A subsequent DSA confirmed a type I dural arteriovenous fistula (DAVF) at the posterior left wall of the foramen magnum fed by the posterior meningeal branch of the vertebral artery (PMA) and branches of the left posterior spinal artery (PSA) - which in our case originated from posterior inferior cerebellar artery (PICA) - and drained by dilated perimedullary veins (Figure 8). After an unsuccessful embolization attempt, the patient underwent a suboccipital craniectomy and C1 laminectomy. The fistula and a flow-related venous aneurysm were coagulated and clipped, respectively. Following intensive care unit (ICU) support, the patient had normal respiratory function and motor improvement, especially in the upper limbs. Though postoperative MRI/MRA showed regression of myelopathy, no residual fistula and deflated veins, and DSA (Figure 9) confirmed the complete fistula obliteration, the patient presented vasodilation due to autonomic failure, which was initially controlled with norepinephrine and later with midodrine. The patient had a prolonged ICU hospitalization and deceased after four months.

Case 3

**Figure 10:**
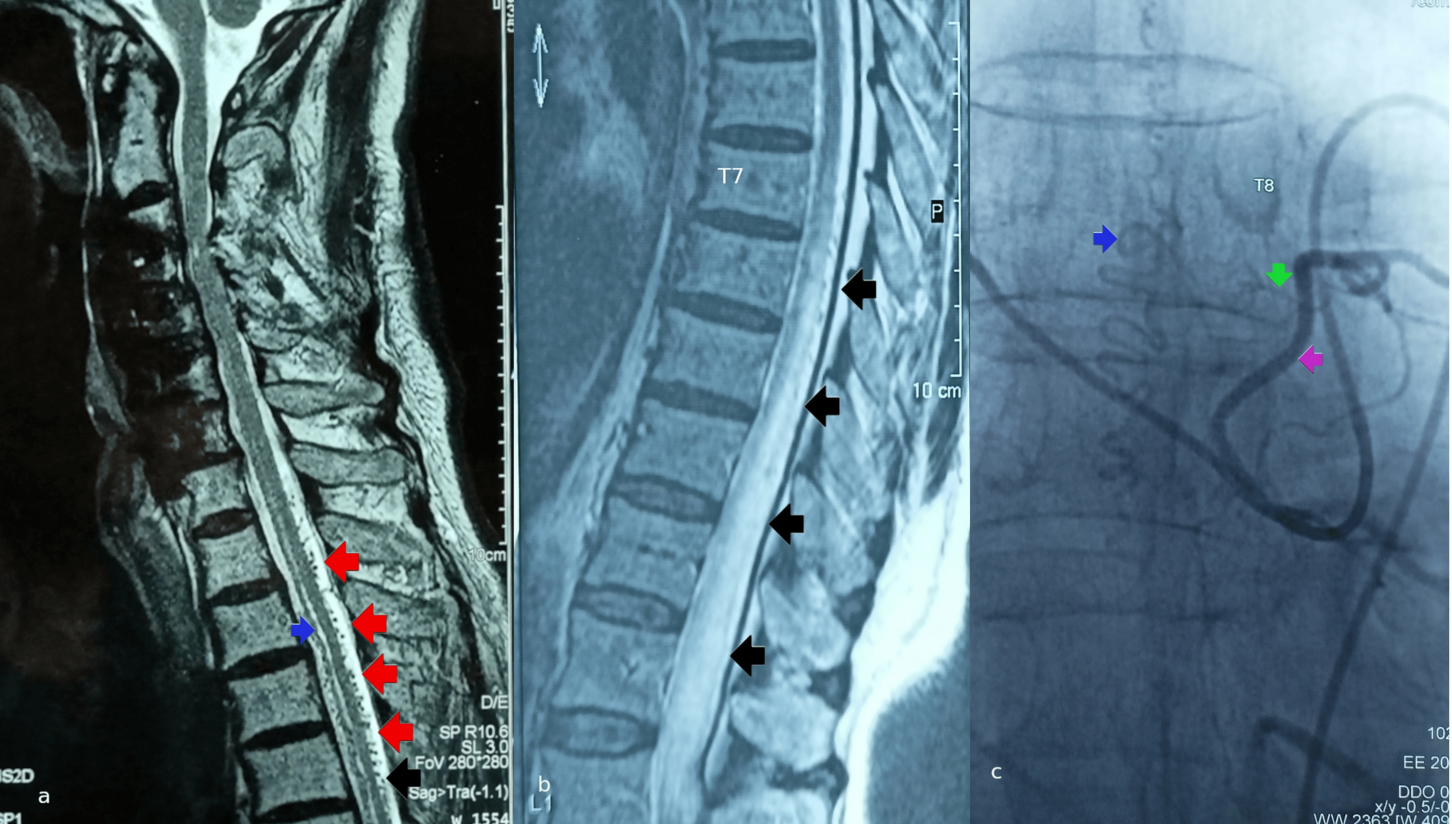
Preoperative imaging of patient #3. a: Cervical T2WI shows intramedullary oedema from Th1 to Th6 (blue arrow), tortuous perimedullary dorsal venous plexus starting from Th1 down to Th6 (red arrows) and the anterior cervical disc fusion already known from patients history (at levels C3-4 until C6-7). b: Thoracic T2WI shows similarly the intramedullary oedema from  Th7 to L1 (arrows) and the congested dorsal perimedullary venous plexus. c: Anteroposterior (A-P) view of the digital subtraction angiography (DSA) shows the segmental Th8 artery (magenta arrow), the fistula between the radicular artery (RA) and radicular vein (green arrow) and the congested posterior spinal vein (blue arrow).

A 74-year-old male with a 2-year history of gait deficit that progressively evolved to non-ambulance, sitting difficulty, and bladder-bowel dysfunction with a permanent bladder catheter. The physical examination revealed paraplegia (all muscle groups had muscle strength MRC 1/5) with right hip and knee stiffness, positive left Babinski reflex, normal patellar and absent Achilles reflex, reduced sphincter tone, absent bulbocavernosus reflex and deep and superficial sensation deficit at Th10 level bilaterally extending to both legs. Electromyography (EMG) showed a myelopathic deficit of both quadricepites and bilateral neurogenic/radicular signal in gastrocnemii and tibialis anteriores (the second suggestive of degenerative foraminal compression or bedriddenness). MRI (Figure 10a, 10b) showed medullary oedema and cord enlargement extending from Th8 until the conus, dorsal perimedullary flow voids on sagittal T2WI and disc disease at L2-3 and L4-5. The DSA showed a left Th8 fistula (Figure 10c). The patient underwent three three-level laminectomy (Th8-10) which disclosed the tortuous RV along the Th9 root sleeve. Its proximal end was cauterized, following the visually confirmed decongestion of the draining vein after temporal clipping of the feeder. The patient’s functional status did not change.

Case 4

**Figure 11:**
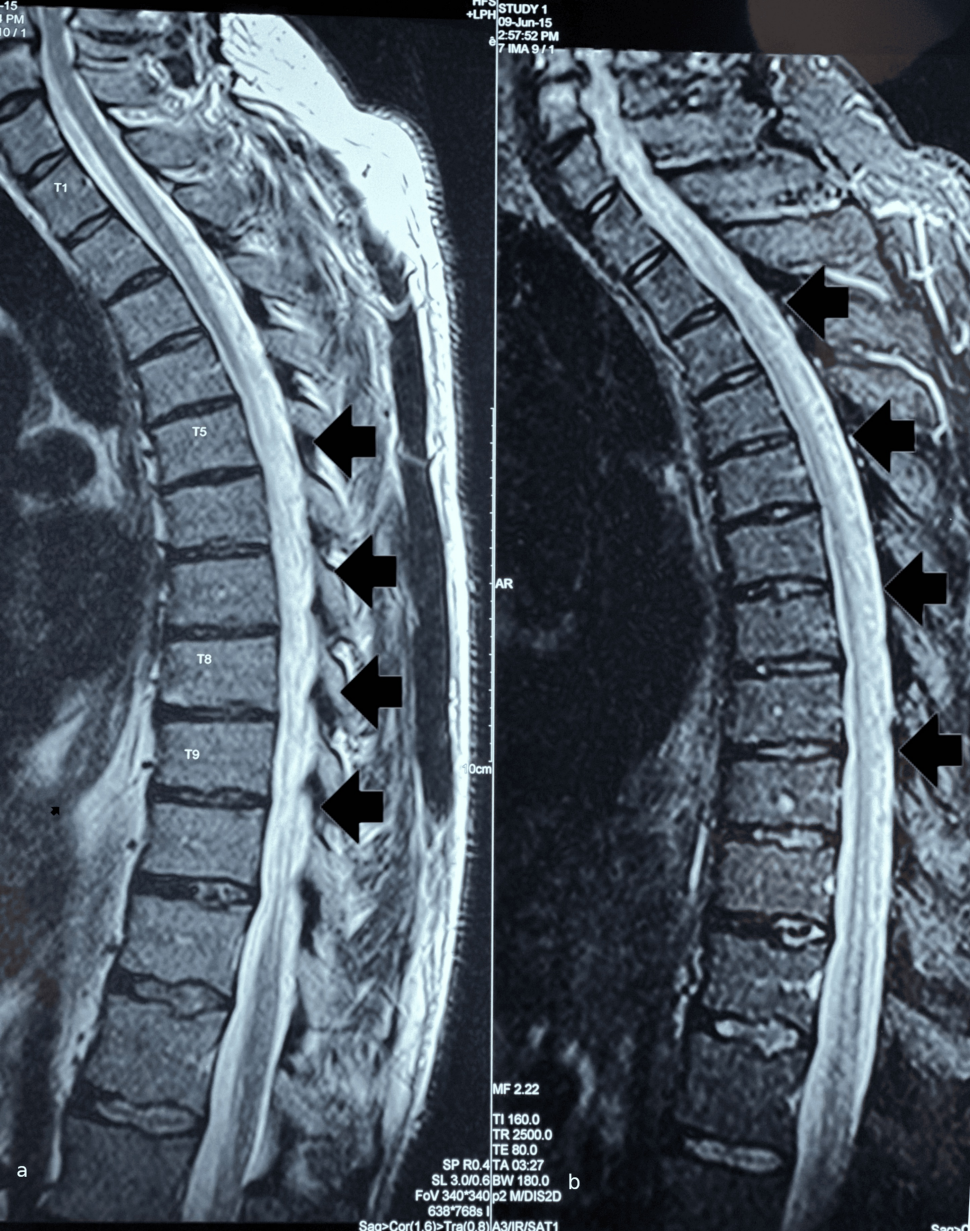
Sagittal T2WI of patient #4. a: Residual congestive myelopathy with indistinguishable borders, at levels Th5-Th10 3 years post-treatment. b: same findings parasagitally.

A 37-year-old male with a history of twice embolized sDAVF at L1 (2.5 years before in the USA and one year before in our hospital) presented with established spastic paraplegia (incomplete ASIA B at Th11 level) at the natural rehabilitation department for regular follow-up with MRI and DSA and management of his spasticity (as high as 3 assessed by the Ashworth scale, bilaterally) and his need for intermittent bladder catheterizations. The DSA confirmed that no recurrence had occurred and the MRI showed the already known residual congestive myelopathy at T2WI, without enhancement at T1WI PG, which had not subsided since treatment (Figure 11). He was discharged after 1 month of regular physiotherapy with unchanged clinical status, unimproved micturition deficits, and bilateral lower limb spasticity. Upon 3-month follow-up his clinical status was still unimproved.

## Discussion

**Figure 12:**
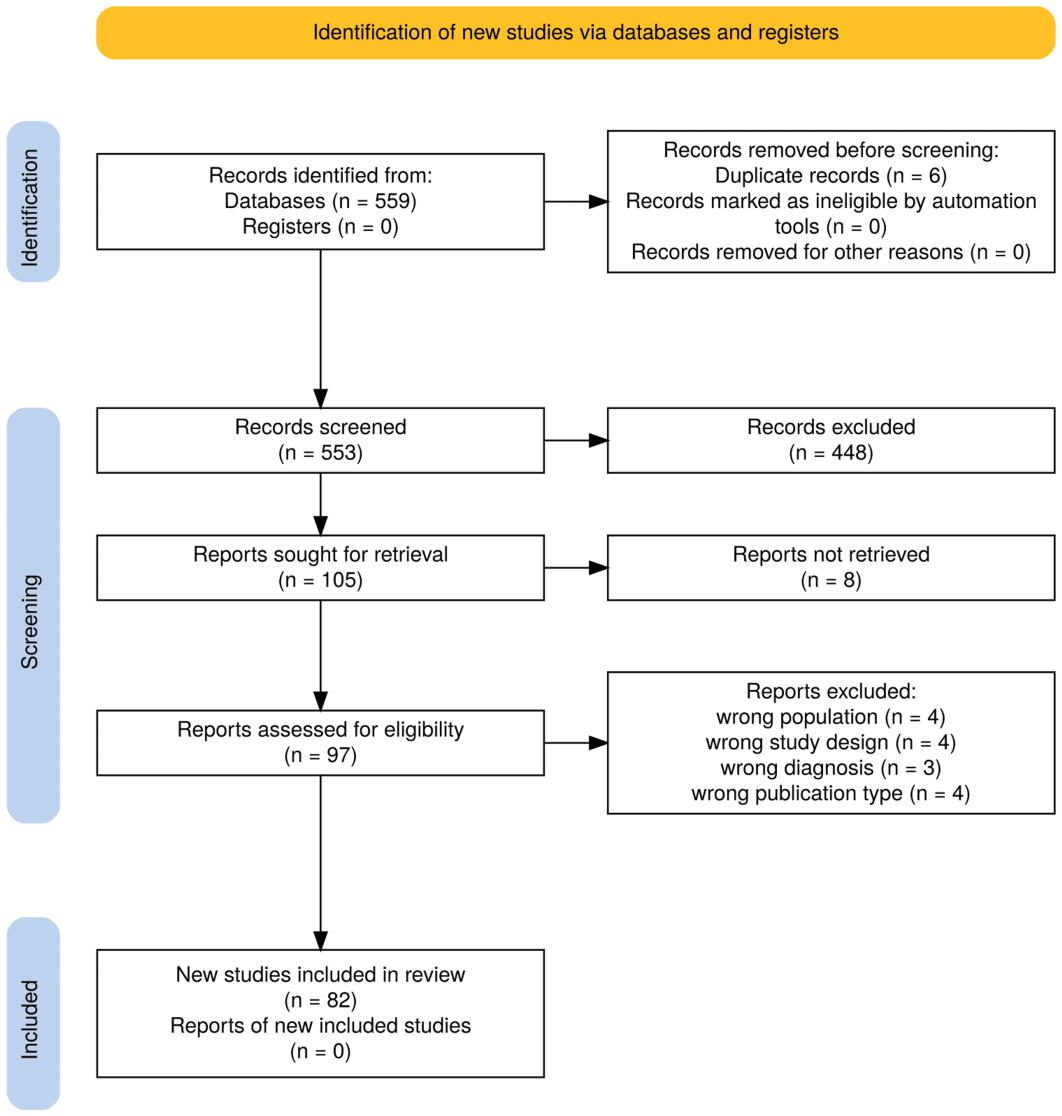
PRISMA 2020 flow chart of study selection PRISMA: Preferred Reporting Items for Systematic Reviews and Meta-Analyses

For this systematic review we adhered to the guidelines of the Preferred Reporting Items for Systematic Reviews and Meta-Analyses (PRISMA) [7]. We searched PubMed with the following keywords, combined with the AND boolean operation: “Spinal”, “Dural”, “Arteriovenous” and “Fistula”, on 6 December 2023. Then we limited our search to the last decade (January 2014 - December 2023). The article selection process involved independent researchers conducting comprehensive searches (Figure 12). The inclusion criteria were original studies (either controlled or observational), case series with five or more patients, an adult population, and a language known to at least one of the authors, i.e., English, German, French, and Spanish. The exclusion criteria were wrong publication type (case reports, case series with less than five subjects, reviews, letters, abstracts, book chapters), wrong population (pediatric, cadaveric, main subject other than sDAVFs, mixed data, non-established techniques), any other language different from English, German, French or Spanish, wrong diagnosis (diagnosis other than sDAVFs and diagnosis of cranial DAVFs), wrong study design (technical note, wrong intervention, without any intervention-related results). In addition, we retrospectively found the medical files of four patients who underwent surgery in our department during the same 10-year period. We reviewed their files, imaging studies, operative reports, and follow-ups. We present them hereafter as illustrative cases of our mini-case series.

**Figure 13:**
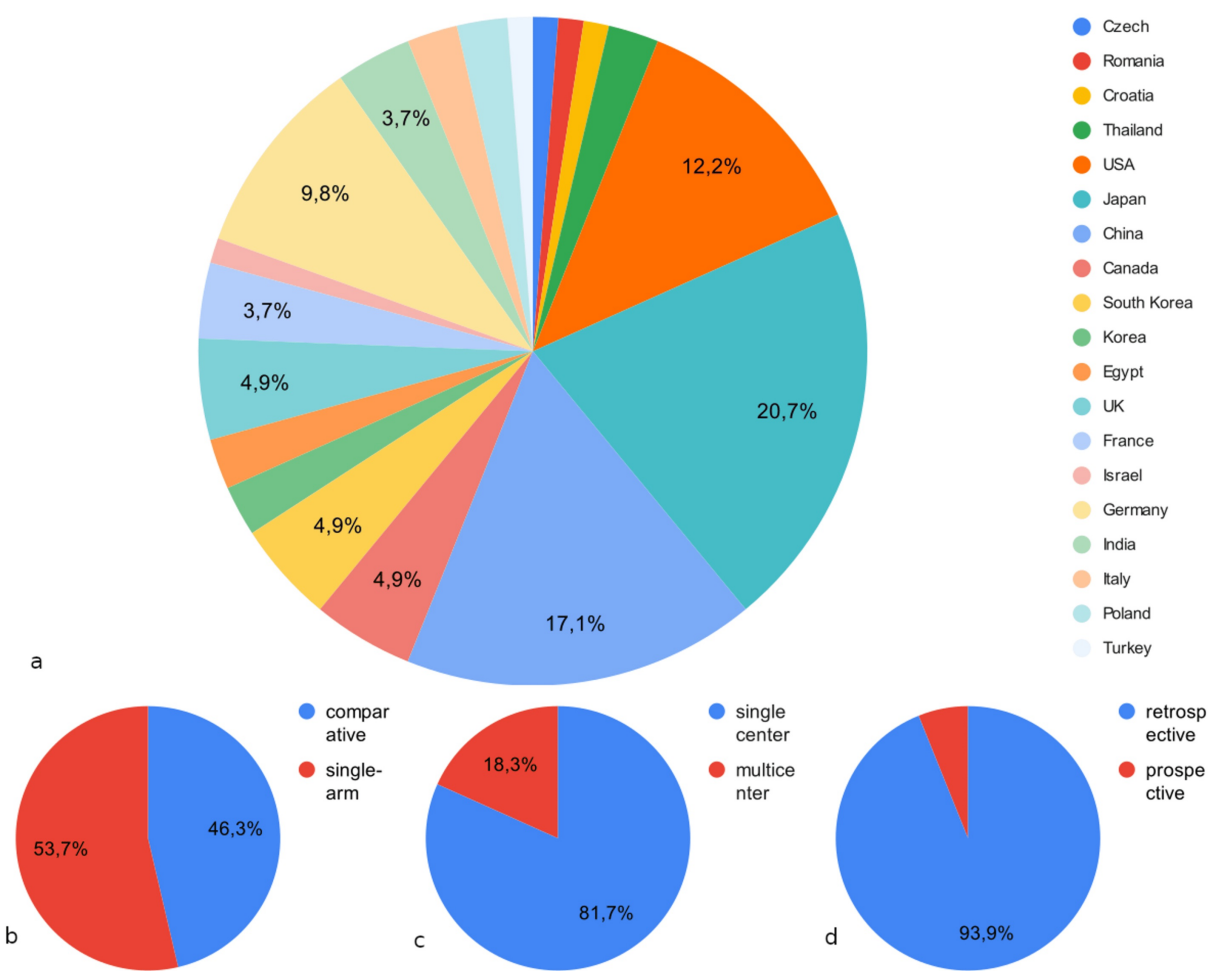
Literature description. a: Percentages of studies contributions with respect to country of origin, b: comparative versus single-arm, c: single-center versus multicentric, d: retrospective versus prospective.

A total of 559 studies were found. These studies were inserted into the Rayyan online artificial intelligence (AI) tool [8] which detected six duplicate articles and automatically generated labels for 281 studies of the remaining 553. We validated the labels of those studies through title and abstract manual screening and excluded them. To provide a more comprehensible report, we manually grouped them under umbrella-term groups, resulting in 270 studies grouped as wrong publication type (216 case reports, 62 reviews, 17 letters, six abstracts, one book chapter), two as wrong population (pediatric population) and nine studies as wrong language. Subsequently, the remaining 272 unlabeled studies were screened manually by title and abstract, independently by two authors. The differences in selection were resolved by a third author. In this step, we manually excluded, in a hierarchical manner, 167 studies (52 as wrong publication type, 71 as wrong population, nine as wrong language, 23 as wrong diagnosis, and 12 as wrong study design). We ended up with 105 studies, of which eight could not be retrieved. We included 97 papers for full-text review, out of which we further excluded 15 studies (four due to wrong population, four due to wrong study design, four due to wrong publication type, and three due to wrong diagnosis). The literature consisted of 82 studies from 19 countries; 76 were retrospective, 35 were comparative, and 66 were single-center (Figure 13). It was not uncommon for a study to report mixed data on sDAVFs and sEAVFs, in which case, we isolated only information regarding sDAVFs. Each study group's age was reported either as mean or median. The aggregate estimate of the mean in the present study is approximated simply as the weighted (by the population of study) average of the study-reported mean or median.

Epidemiologic data

Epidemiological data were retrieved from seventy-four studies, considering that age (mean or median) and gender clearly referred to sDAVF patients. Therefore, a total of 3130 patients had a mean weighted age of 61 years (95%CI: 59.5-62.4) and male prevalence (2467 patients, 78.8%). The fistula level was reported clearly only in 64 studies (cervical: 329 sDAVFs, 11.9%; thoracic: 1791 sDAVFs, 65%; lumbosacral: 632 sDAVFs, 22.9%). Only three [9-11] studies reported multiple fistulas. Moreover, the mean delay time from symptom onset to definite diagnosis was calculated from 51 studies to be 14.7 months (95%CI: 14.1-15.2) (Table 1).

**Table 1:** Epidemiological data. M: male, C: cervical, T: thoracic, L: lumbar, S: sacral. *:studies that reported multiple sDAVFs, that is, more sDAVFs than patients, -: missing or no data.

Reference	Country	Period	No. of sDAVF patients	Mean (or median) age (years)	Gender (M)	Location (C)	Location (T)	Location (L&S)	Delay to diagnosis (months)	Delay to diagnosis (months)
Wang et al., 2023 [12]	China	2002 - 2020	65	57,4	58	65	0	0	7,2	7,2
Zhu et al., 2023 [13]	China	2016 - 2021	38	66	23	0	0	38	5	5
Peng et al., 2023 [14]	China	1/2013 - 09/2021	181	56,8	104	63	79	39	14,5	14,5
Szmygin et al., 2023 [15]	Poland	1/2014 - 12/2020	16	62,4	12	0	16	0	13,3	13,3
Ouyang et al., 2023 [16]	China	1/2018 - 1/2022	28	56,6	22	2	26	0	-	-
Mundhe et al., 2023 [17]	India	1/2009 - 12/2020	8	51	5	8	0	0	-	-
Inoue et al., 2023 [18]	Japan	2009 - 2019	14	59	10	14	0	0	8,3	8,3
Iampreechakul et al., 2023 [19]	Thailand	6/2018 - 12/2021	7	52,7	5	7	0	0	-	-
Devalckeneer et al., 2023 [20]	France	3/2013 - 3/2020	21	56,2	15	0	16	5	-	-
Choi et al., 2023 [21]	South Korea	3/2013 - 2/2021	16	56,9	12	16	0	0	38,9	38,9
Boonyakarnkul et al., 2023 [22]	Thailand	1/2006 - 10/2020	31	61	25	2	20	9	8,5	8,5
Zhang et al., 2022 [10]	China	6/2013 - 1/2016	32	59,1	29	6	19	7	14,7	14,7
Yang et al., 2022 [23]	China	2013 - 2021	22	61,9	18	1	17	4	12,2	12,2
Yang et al., 2022 [24]	China	3/2013 - 12/2014	86	53	71	0	72	14	12,8	12,8
Ozpeynirci et al., 2022 [25]	Germany	12/2011 - 1/2021	25	65	19	-	-	-	-	-
Naamani et al., 2022 [26]	USA	11/2003 - 11/2021	27	67,8	23	1	20	6	-	-
Luo et al., 2022 [27]	China	1/2013 - 6/2020	76	56	64	0	50	26	-	-
Hiramatsu et al., 2022 [28]	Japan	4/2009 - 3/2019	31	68	25	14	15	2	8	8
Vukic et al., 2021 [29]	Croatia	1.2009 - 1/2020	34	65,5	21	1	18	8	12	12
Tanaka et al., 2021 [30]	Japan	2005 - 2017	22	67	21	-	-	-	-	-
Rothrock et al., 2021 [31]	USA	1/1997 - 12/2020	5	63	4	0	4	1	5	5
Oh et al., 2021 [32]	Korea	1/2004 - 6/2017	38	59	30	5	24	9	26	26
Luo et al., 2021 [33]	China	1/2013 - 6-2020	76	56	64	0	50	26	14	14
Lee et al., 2021 [34]	Korea	1/2004 - 3/2019	71	59,5	60	11	45	15	-	-
Zhang et al., 2020 [35]*	China	1/2009 - 4/2018	65	53,5	52	5	45	18	20,7	20,7
Takai et al., 2020 [36]	Japan	2009 - 2018	195	66	161	0	158	37	20	20
Ronald et al., 2020 [11]*	USA	2006 - 2018	46	63	35	4	28	15	16,8	16,8
Murphy et al., 2020 [37]	USA	2008 - 2018	57	64	49	7	29	21	-	-
Jablawi et al., 2020 [38]	Germany	1990 - 2018	19	65	16	0	0	19	15	15
Du et al., 2020 [39]	China	2004 - 2018	79	59	67	2	56	21	7	7
Gogu et al., 2020 [40]	Romania	2012 - 2017	11	52,6	12	0	7	4	3	3
Yamahata et al., 2019 [41]	Japan	2005 - 2017	20	64	18	0	10	10	-	-
Tsuruta et al., 2019 [42]	Japan	2007 - 2009 , 2010-2014	172	62,5	133	24	148	0	14,9	14,9
Takai et al., 2019 [43]	Japan	6/2002 -7/2018	40	67	33	2	29	9	9,4	9,4
Shimizu et al., 2019 [44]	Japan	-	20	63,5	15	7	7	6	-	-
Kannath et al., 2019 [45]	India	2007 - 2017	32	55,4	27	-	-	-	-	-
Jablawi and Mull et al., 2019 [46]	Germany	6/2006 - 3/2017	40	70	28	0	26	14	20,2	20,2
Jablawi and Mull et al., 2019 [47]	Germany	1997 - 2017	3	67,9	3	-	-	-	-	-
Fiaschi et al., 2019 [48]	Italy	4/2007 - 3/2017	19	64,4	14	0	17	2	6	6
Bretonier et al., 2019 [49]	France	2000 - 2017	63	65,7	48	-	-	-	15,3	15,3
Akgun et al., 2019 [50]	Turkey	1999 - 2018	46	54,7	37	0	36	10	21,4	21,4
Sultan et al., 2018 [51]	Egypt	2006 - 2014	13	56	12	0	8	5	6,46	6,46
Safaee et al., 2018 [9]*	USA	1997 - 2018	41	64	30	2	30	12	14	14
Ma et al., 2018 [52]	China	3/2013 - 12/2014	94	53,5	78	0	75	19	12,7	12,7
Jablawi et al., 2018 [53]	Germany	6/2010 - 2/2016	53	67,5	38	-	-	-	19,8	19,8
Hunt et al., 2018 [54]	UK	11/2006 - 11/2017	37	62	28	1	26	10	9,4	9,4
Hiramatsu et al., 2018 [55]	Japan	2000 - 2015	22	69	18	22	0	0	-	-
Endo et al., 2018 [56]	Japan	2008 - 2015	25	68	20	0	16	9	14,2	14,2
Cesak et al., 2018 [57]	Czech	2007 - 2017	24	60,5	18	1	16	7	12	12
Wojciechowski et al., 2017 [58]	Poland	-	17	61	14	0	13	4	18	18
Sasamori et al., 2017 [59]	Japan	1995 - 2017	33	70	25	4	22	7	19,2	19,2
Fox et al., 2017 [60]	Canada	2004 - 2011	10	62,6	7	0	9	1	55	55
Koch et al., 2017 [61]	USA	1/2004 - 12/2013	34	63,3	29	2	20	12	-	-
Kiyosue et al., 2017 [3]	Japan	2005 - 2016	108	64,4	89	0	94	14	-	-
Gross et al., 2017 [62]	USA	1/1998 - 10/2015	71	63	55	4	45	22	-	-
Durnford et al., 2017 [63]	UK	1992 - 2014	59	63	47	3	37	19	9	9
Adrianto et al., 2016 [64]	South Korea	1993 - 2015	9	55,3	8	2	6	1	15	15
Zogoropoulos et al., 2016 [65]	Japan	2009 - 2014	14	62,1	12	2	10	2	13,5	13,5
Sasamori et al., 2016 [66]	Japan	1995 - 2011	50	63,2	38	7	31	12	19,7	19,7
Lee et al., 2016 [67]	South Korea	1992 - 2014	39	58,2	27	4	24	11	10,2	10,2
Kuwayama et al., 2016 [68]	Japan	1998-2002	100	58,1	72	-	-	-	-	-
Brinjikji et al., 2016 [69]	USA	2000 - 2014	53	65	43	-	-	-	9,2	9,2
Sri et al., 2015 [70]	UK	1997 - 2010	38	63,5	27	0	38	0	-	-
Shin et al., 2015 [71]	South Korea	2002 - 2007	15	48	13	0	12	3	15	15
Schuss et al., 2015 [72]	Germany	1990 - 2012	29	61	26	0	20	9	21	21
Ozkan et al., 2015 [73]	Germany	1/2001 - 12/2013	32	64	26	1	22	9	18,1	18,1
Iovtchev et al., 2015 [74]	Israel	1992 - 2012	7	60,3	7	-	-	-	10	10
Chibbaro et al., 2015 [75]	France	1/2001 - 12/2008	30	62	25	0	16	14	27	27
Yen et al., 2014 [76]	UK	2003 - 2013	12	53	8	3	5	4	-	-
Rashad et al., 2014 [77]	Egypt	2006 - 2013	12	56	11	0	7	5	-	-
Qi et al., 2014 [78]	China	2008 - 2013	52	59,6	43	1	38	13	14,4	14,4
Lindenholtz et al., 2014 [79]	Canada	1999 - 2012	53	60,6	49	1	42	10	-	-
Gokhale et al., 2014 [80]	USA	1993 - 2012	27	57	20	2	22	3	12,72	12,72
Cao et al., 2014 [81]	China	2006 - 2011	20	58	16	-	-	-	10	10
			3130	60,97 (59,5 - 62,4)	2467 (78,8%)	329 (11,9%)	1791 (65%)	632 (22,9%)	14,7 (14,1 - 15,2)	14,7 (14,1 - 15,2)

According to aggregate data, the typical patient profile is male, in his mid-60s, with symptoms lasting 6 to 12 months before a diagnosis can be established. The experience from our department does not deviate from this patient profile as all four cases were male. Presenting symptoms and functional status usually deteriorate during the definite-diagnosis delay period - from initial presentation to time of diagnosis - and most frequently include motor weakness, varying from simple paresis to plegia, as well as sensory and bladder or bowel disturbances. Though symptoms progress slowly in the usual thoracolumbar sDAVF, progression can be acute in the rarer case of cervical lesion [82]. Overall clinical status varied and was assessed with the modified Aminoff and Logue's Scale (mALS), modified McCormick Scale (mMCS), Nurick scale, and modified Rankin scale (mRS), while one study also used the modified Dennis scale (mDS) to assess pain. Common imaging studies include MRI and DSA. According to those routine imaging studies, the most typical findings on sagittal T2WI were dorsal flow voids and intramedullary edema, both extending throughout multiple levels. The most common fistula location was at the thoracolumbar level, unilateral, without side preference, and had a single feeder.

Prognostic factors

Overall, sDAVF patients have a worse prognosis [40] compared with other causes of myelopathy, and their quality of life is lower than the population average [59] even post-treatment with low potential for ambulation [74]. A lot of controversy with respect to the statistical significance of prognostic factors, for sDAVF patients specifically, remains unresolved. Thirty-three studies were able to establish the statistical significance of prognostic factors, 14 could only show correlation, while 35 were irrelevant to that subject. The following prognostic factors still remain subject to controversy: 1. preoperative clinical status (16 studies [10,14,23,33,37,39,42,48,49,51,60,67,71,73,76,77] showed statistically significant prognostic value versus nine studies [9,12,32,34,35,57-59,69,83] could not establish statistical significance); 2. delay to final diagnosis or initial misdiagnosis (10 [11,24,36,37,43,48,50,51,60,80] versus 12 [9,12,23,32,34,35,52,57-59,61,63,65,72-74] studies); 3. age (three [12,73,77] versus nine [23,32,35,51,52,57-59,65] studies). Other variables regarding the patient profile, such as comorbidities [73], presence of bladder-bowel disturbances (BBD) [70], and acute onset of symptoms [42,46,72], though only scarcely reported in the literature, whenever studied, statistical significance was shown. More controversy exists around the prognostic value of imaging findings, such as pre- and postoperative extent of intramedullary edema (four [29,33,67,71] versus five [9,35,39,49,72] studies), the anterior spinal artery (ASA)-related feeder origin (one [64] versus one [32] study) or the extent of flow voids (one [33] versus one [27] study). Cranial flow of drainage does not seem to hold any prognostic value. As one would expect, intervention-related events, such as complication occurrence, initial intervention failure, and the necessity of both intervention modalities and recurrence, affect the clinical outcome. The prognostic role of the caudal location of the fistula has not been established [9,38], but some trends have been reported. Zhang et al [35] showed a trend of better outcomes for lower thoracic and lumbosacral locations when compared with craniocervical junction (CCJ) and cervical. Jablawi et al [38] reported that lumbosacral sDAVFs, when compared to thoracic, have a worse prognosis. However, a comparative study by Endo et al [56] showed that micturition had a higher improvement rate in the lumbosacral group compared to the thoracic group. None of the studies could establish any difference in clinical outcome between the two modalities, except for Česák et al, who showed that although the improvement rate is the same, the surgical option had better 6-month mRS improvement than embolism [57].

A discrepancy between clinical outcome and successful obliteration rate was observed for spinal sDAVFs, while for their cranial counterpart, the two variables correlate [68]. This has been attributed to the diagnosis delay, which is common in spinal but not in cranial DAVFs [68]. The overall prognosis regarding the natural history and postoperative quality of life for sDAVFs is poor [59,68]. The accumulated evidence on most prognostic factors cannot lead to consensus. Even preoperative clinical status generated much controversy in our review, with 33 studies proving statistical significance and 14 studies only able to show a trend. Only one of our patients achieved baseline clinical status without any severe deficit (case 1), while both patients (cases 3-4) with progressed status did not show any improvement.

Imaging

It should be emphasized that lumbar puncture, steroids, or inappropriate surgery should be postponed until the diagnosis of sDAVF is excluded, as these procedures can worsen the patient’s clinical status [24]. sDAVFs are diagnosed and localized by DSA, which is the gold standard, after indirect findings on the MRI - e.g., snake eyes sign and missing piece sign [12,13]. Out of the 22 studies that focused on the diagnostic value of imaging and the localizing procedure, four studies discussed specific DSA technical issues [3,21,22,36] and seven addressed the usefulness of the preoperative MRI to guide DSA [16,25,26,30,79,84,85] (five of them confirmed that preoperative MRI can guide the DSA). Double sDAVFs are extremely rare, with only 40 cases in the bibliography [9,35,36,46], either synchronously or metachronously, the majority of which were found within three vertebral levels [47]. Even when the lesion is single, which is the most common finding, the feeders can be multiple (153 out of 428 patients, that is 35.7% in seven studies) and usually originate from different levels [22]. The false negative rate of DSA can reach up to 8.3% (39 out of 469 patients in nine studies). Moreover, newer MR sequences such as Time Resolved Imaging of Contrast Kinetics (TRICKS) [26,84] or sampling-perfection with application-optimized-contrast using different flip-angle evolution (SPACE) [3,16] have been developed. Preoperative MR scans can reduce the number of levels to be explored during the DSA [84] and shorten the DSA duration [85]. Recently, Ouyang et al. showed that the combination of SPACE(Sampling Perfection with Application-optimised Contrasts using different flip-angle Evolutions)-MR and contrast-enhanced (CE)-MRA has an accuracy similar to DSA [16], validating the similar earlier finding about CE-MRA by Lindenholtz et al. [79]. In addition, Naamani et al. showed that TRICKS has a within-2-level localization positive predictive value (PPV) of 75% [26]. However, there is still controversy on how reproducible these results are [25,30].

Intervention

Α total of 1837 patients of the aggregate patient population underwent surgery, whereas 1085 embolism. Surgery was the primary therapy in 30 studies and embolism in nine, whilst only 16 studies compared the two modalities, either directly or indirectly, by performing statistical comparison between the two modalities or splitting the patient population proportionately. Twenty-three studies were irrelevant to intervention-related events.

We collected data from studies that clearly stated the intervention-related events in their results. Herein we report aggregate patient data. In the 56 studies that explicitly reported the occlusion outcome as validated by DSA, the incomplete obliteration rate for surgery and embolism was 1.9% (23 out of 1213 patients in 50 studies) and 28.9% (269 out of 932 patients in 42 studies), respectively. The complication rate when vascular-related events were considered, was higher for embolism (6.8% in studies = 22 vs for surgery 0.8% in 28 studies) (Table 2). Additionally, regarding the surgery-related complications, cerebrospinal fluid (CSF)-leak was observed in 1.8%; meningocele in 0.8%; spinal epidural or subdural hematoma in 1.8%, and wound infection in 1.6% (Table 2). The recurrence rate for surgery and embolism was calculated at 1.9% (19 out of 1012 in 34 studies) and 9.6% (63 out of 655 in 30 studies), respectively (Table 3).

**Table 2:** Intervention-related data: complete occlusion and complication rates. Infarct: infarction, Maj a occl: major spinal artery occlusion, Vasc inj: vascular injury, CSF leak: cerebrospinal fluid leakage, -: missing or no data.

Reference	Surgery							Embolism		
	Surgery (first intervention)	Complete obliteration after first surgery	Complications					Embolism (first intervention)	Complications	
			Infarct, Maj a occl, Vasc inj	CSF leak	Meningocele	Hematoma	Wound infection		Complete obliteration after first embolism	Infarct, Vasc inj
Takai et al., 2023 [82]	24	22	2	2	0	2	0	3	1	1
Szmygin et al., 2023 [15]	-	-	-	-	-	-	-	16	10	1
Mundhe et al., 2023 [17]	-	-	-	-	-	-	-	8	8	1
Inoue et al., 2023 [18]	10	10	2	0	0	0	0	3	3	0
Iampreechakul et al., 2023 [19]	6	6	0	0	0	0	0	1	0	-
Devalckeneer et al., 2023 [20]	12	11	0	0	0	0	0	-	-	-
Choi et al., 2023 [21]	4	4	0	0	2	0	0	12	1	2
Boonyakarnkul et al., 2023 [22]	-	-	-	-	-	-	-	28	24	3
Zhang et al., 2023 [10]	32	32	-	-	-	-	-	-	-	-
Yang et al., 2022 [24]	75	75	-	-	-	-	-	9	7	-
O Reilly et al., 2022 [86]	2	1	-	-	-	-	-	2	1	-
Oh et al., 2021 [32]	4	4	0	0	0	0	0	34	16	5
Lee et al., 2021 [34]	15	15	-	-	-	-	-	56	37	4
Zhang et al., 2020 [35]	65	65	0	0	0	0	1	-	-	-
Takai et al., 2020 [36]	145	144	0	1	0	0	1	50	40	2
Ronald et al., 2020 [11]	40	37	-	-	-	-	-	7	6	-
Murphy et al., 2020 [37]	21	17	-	-	-	-	-	33	30	-
Jablawi et al., 2020 [38]	19	19	0	1	0	0	0	-	-	-
Gogu et al., 2020 [40]	32	32	-	-	-	-	-	-	-	-
Yamahata et al., 2019 [41]	18	18	-	-	-	-	-	2	2	-
Tsuruta et al., 2019 [42]	-	-	-	-	-	-	-	136	74	9
Takai et al., 2019 [43]	40	40	-	-	-	-	-	-	-	-
Shimizu et al., 2019 [44]	5	5	-	-	-	-	-	5	5	0
Jablawi and Mull et al., 2019 [46]	40	39	0	1	0	0	1	-	-	-
Jablawi and Mull et al., 2019 [47]	3	3	-	-	-	-	-	-	-	-
Fiaschi et al., 2019 [48]	18	18	-	-	-	-	-	-	-	-
Bretonier et al., 2019 [49]	23	21	0	1	0	2	1	40	28	1
Akgun et al., 2019 [50]	14	14	-	-	-	-	-	25	18	-
Sultan et al., 2018 [51]	11	11	0	1	0	0	0	2	1	-
Safaee et al., 2018 [9]	35	34	-	-	-	-	-	6	1	-
Ma et al., 2018 [52]	81	81	0	0	0	1	1	13	11	-
Jablawi et al., 2018 [53]	53	52	0	1	0	0	0	-	-	-
Cesak et al., 2018 [57]	8	7	0	0	1	2	0	16	10	0
Wojciechowski et al., 2017 [58]	14	14	0	2	0	3	0	-	-	-
Sasamori et al., 2017 [59]	14	14	-	-	-	-	-	19	13	-
Fox et al., 2017 [60]	9	9	0	0	1	0	0	-	-	-
Koch et al., 2017 [61]	14	14	2	0	1	0	0	17	11	-
Kiyosue et al., 2017 [3]	39	39	0	0	0	0	1	47	44	4
Kannath et al., 2017 [85]	7	7	-	-	-	-	-	-	-	-
Gross et al., 2017 [62]	43	43	0	3	0	1	2	28	15	2
Durnford et al., 2017 [63]	37	37	0	0	1	0	1	22	12	2
Adrianto et al., 2016 [64]	5	3	-	-	-	-	-	4	3	-
Zogoropoulos et al., 2016 [65]	3	3	0	1	0	0	0	10	8	0
Sasamori et al., 2016 [66]	19	18	0	0	0	2	1	31	25	3
Lee et al., 2016 [67]	5	5	0	0	0	0	0	28	20	0
Sri et al., 2015 [70]	2	2	-	-	-	-	-	36	18	0
Schuss et al., 2015 [72]	29	29	0	0	0	0	0	-	-	-
Ozkan et al., 2015 [73]	25	25	-	-	-	-	-	5	1	-
Iovtchev et al., 2015 [74]	3	1	-	-	-	-	-	4	1	-
Chibbaro et al., 2015 [75]	22	22	0	0	0	0	0	120	118	-
Yen et al., 2014 [76]	2	2	-	-	-	-	-	10	8	2
Rashad et al., 2014 [77]	9	9	-	-	-	-	-	2	1	-
Qi et al., 2014 [78]	40	40	0	0	0	1	1	12	7	-
Gokhale et al., 2014 [80]	17	17	0	0	0	0	2	10	10	1
Blackburn et al, 2014 [87]	-	-	-	-	-	-	-	20	14	-
	1213	1190 (98,1%)	6 (0,75%)	14 (1,75%)	6 (0,75%)	14 (1,75%)	13 (1,63%)	932	663 (71,13%)	43 (6,83%)

**Table 3:** Recurrence rates. -: absent or missing data.

Reference	Surgery (fisrt intervention)	Embolism (first intervention)	Recurrence (surgery)	Recurrence (embolism)
Wang et al., 2023 [12]	57	8	4	5
Vercelli et al., 2023 [83]	16	13	0	6
Szmygin et al., 2023 [15]	-	16	-	2
Mundhe et al., 2023 [17]	-	8	-	0
Choi et al., 2023 [21]	4	12	0	2
Zhang et al., 2022 [10]	32	-	0	-
Yang et al., 2022 [23]	75	9	0	1
Luo et al., 2022 [27]	76	-	0	-
Vukic et al., 2021 [29]	24	10	1	1
Oh et al., 2021 [32]	4	34	0	5
Lee et al., 2021 [34]	15	56	0	8
Zhang et al., 2020 [35]	65	-	3	-
Takai et al., 2020 [36]	145	50	0	8
Ronald et al., 2020 [11]	40	7	1	0
Jablawi et al., 2020 [38]	19	-	5	-
Du et al., 2020 [39]	34	38	0	5
Takai et al., 2019 [43]	40	-	0	-
Shimizu et al., 2019 [44]	5	5	0	0
Fiaschi et al., 2019 [48]	18	-	0	-
Bretonier et al., 2019 [49]	23	40	6	6
Safaee et al., 2018 [9]	35	6	0	1
Ma et al., 2018 [52]	81	13	0	1
Cesak et al., 2018 [57]	8	16	0	6
Wojciechowski et al., 2017 [58]	14	-	1	-
Kiyosue et al., 2017 [3]	39	47	0	3
Kannath et al., 2017 [85]	7	-	1	-
Gross et al., 2017 [62]	43	28	0	0
Durnford et al., 2017 [63]	37	22	1	2
Zogoropoulos et al., 2016 [65]	3	10	0	0
Sasamori et al., 2016 [66]	19	31	0	3
Lee et al., 2016 [67]	5	28	0	0
Schuss et al., 2015 [72]	29	-	0	-
Ozkan et al., 2015 [73]	25	5	0	0
Chibbaro et al., 2015 [75]	22	120	0	2
Rashad et al., 2014 [77]	9	2	0	1
Qi et al., 2014 [78]	40	12	0	1
Gokhale et al., 2014 [80]	17	10	0	3
Blackburn et al., 2014 [87]	-	20	-	2
	1052	655	19 (1,87%)	63 (9,61%)

A certain number of studies found significant differences when compared between the two modalities. Seven studies [19,21,32,36,49,57,61] prove surgery’s higher initial complete obliteration rate, while also eight studies [12,36,39,49,57,78,80,83] prove surgery’s lower recurrence rate. None supports the contrary. Five studies [59,61,63,65,80] showed that clinical outcome is independent of intervention modality, whereas in nine studies [9,23,37,39,49,51,57,61,80] intervention is not an independent prognostic factor. None reported the contrary, except for Česák et al [57], who, although showed that both groups, surgical and endovascular, had a similar overall therapeutic impact, they noted that the surgery group, in comparison to the endovascular, had a statistically significant long-term continuous improvement effect, at 1 month, 6 months and 1-year follow-up. Finally, nine [12,36,39,49,57,59,62,80,83] out of sixteen comparative studies reported a similar complication rate of the two modalities, which is confirmed by the aggregate data.

Indications and contraindications of embolism

Seventeen studies performed embolism as a first-line treatment. Among those, the most acknowledged - by the authors of the respective study - reasons to preclude a patient from embolism are: 1) ASA, PSA, or direct Adamkeiweicz contribution to the feeder (in nine studies [34,57,59,61,62,64,70,75,87]); 2) feeder morphology that increases the difficulty of vessel catheterization such as tortuosity, small feeder size, atherosclerotic vessel (in seven studies [32,34,59,62,70,75,87]); 3) craniocervical junction (CCJ) or cervical level of the lesion, which is associated to more frequent ASA/PSA contribution to the feeder (in two studies [42,59]) - collateral supply to critical vessels (ASA and VA) is more prominent at the cervical level rendering the risk of stray emboli migrating to those vessels unacceptably high. Generally, a failed embolism or late post-embolism recurrence was almost always followed by surgery. Overall ASA and lateral spinal artery (LSA) contribution rate reached 16.3% (40 out of 244 patients in seven studies [18,32,57,59,61,64,82]). Particularly sDAVFs at CCJ implicate ASA or LSA more frequently than caudal sDAVFs, according to the review by Choi et al. (63% vs 14%), while in their series, this percentage reached 75% [21]. ASA/LSA contribution was shown to complicate the embolism by increasing both the infarction and failed obliteration rates, therefore, surgery is preferred in such cases. Nevertheless, embolism was encouraged in some exceptions, like the presence of bilateral feeders, which after the catheter is positioned protect, through a reversed flow, against the inadvertent advance of the embolic agent to the arterial part of the vessel [64]. This case can also be simulated technically with the flow arrest achieved when the catheter is advanced beyond the origin of the RMedA and radiculopial (RpA) arteries [64]. Five studies did not comment on indications and decided embolism to be the first-line intervention based on institute common practice and experience [15,17,22,67,76].

Embolism as a first option

The surgical option has inarguably higher initial occlusion and lower recurrence rate, and subsequently, the operation-first strategy is more prevalent in the bibliography (36 studies). An embolism-first strategy has also been recommended by multiple authors (17 studies), especially when we consider a patient ineligible for surgery and without contraindications for embolism. It is also logical to consider that since all patients undergo DSA preoperatively, they have the potential for an embolism. The latter is supported by a similar complication rate and clinical outcome to the surgical option. Conversely, one can reasonably wonder how we can suggest embolism when the controversy of delay to treatment remains unresolved and when there exists unequivocal evidence associating failure of initial treatment with worse prognosis. Durnford et al. ask this question in their series, where patients who underwent secondary surgery after a failed embolism had worse gait and urinary outcomes than primary successful intervention, whichever that was (surgery or embolism) [63]. However, Sri et al advocated that it is acceptable to follow an embolism-first strategy after reporting an improvement rate of 74% in such patients [70]. From our point of view, among multiple embolism contraindications, the cervical level of the fistula is still a debate. Out of the 11 studies that focused on cervical and CCJ sDAVFs, six [4,18,19,19,21,28] suggest a surgery-first strategy, whereas only three [17,34,42] an embolism-first strategy. Multiple reasons account for this tendency. More concretely, the recurrence rate for CCJ and cervical sDAVFs was 6.1% for surgery (four out of 66 patients) and 16.8% for embolism (15 out of 89 patients). Similarly, for the initial failure, the respective rates were 3.1% (two out of 64 patients) and 42.4% (95 out of 224 patients), while for vascular-related complications (direct vascular injury or infarction), rates were 0.1% and 2.4%. Given the increased notorious disease progression during the period until definite treatment and the increased rates for both initial failure and recurrence, we can only suggest that more research should be conducted for an embolism-first strategy regarding cervical sDAVFs.

Therapeutic Strategy

Our review does not diverge from the results of multiple recent meta-analyses that compare endovascular and operative interventions. In the seminal meta-analysis by Steinmetz et al [88], occlusion rates of 98% and 46%, complication rates of 1.9% and 3.7%, and morbidity of 2% and 4%, for surgery and embolism, respectively, whereas mortality was zero for both. Overall 89.3% of patients had “no-worse” outcome (54.9% improved), while the same rate for micturition specifically reached 89.4% (33% improved). That meta-analysis did not prove the prognostic value of age, gender, fistula location, or comorbidities, nor did they attribute any outcome difference to one of the two intervention modalities. Though the overall outcome is promising, delayed postoperative deterioration ranging from 6 months to a couple of years has been described quite often [59,63,88] and therefore, should be anticipated.

More recently, Mamaril-Davis et al [89], though they found that the two interventions are similarly popular, confirmed that surgery is therapeutically superior to embolism, despite the recent advancement in embolic agents. Initial occlusion rates for surgery and embolism were 97.7% and 70.6%, and recurrence rates were 2.3% and 13.1%, respectively. Even though they did not compare clinical outcomes between the two modalities, they did prove that both major and minor complication rates were not statistically different between the two modalities (0.8% and 3.4% for surgery and 1.1% and 3.7% for embolism). In addition, Goyal et al [90] found that surgery had significantly better clinical outcomes (odds ratio (OR) = 2.73). They also found an initial occlusion and recurrence OR in favor of surgery (0.15 and 0.18). They, too, found no difference in complication rates. Bakker et al [91] showed successful overall treatment (initial occlusion and no-recurrence) rates were 96.6% for surgery and 72.2% for embolism (recurrence OR was 3.15, in favor of surgery, with embolism reaching a recurrence rate of 31.5%). They did not analyze clinical outcomes and could not compare complication rates due to incomplete and fractionated data.

Our results favor surgery, too, when compared with embolism (incomplete fistula obliteration, recurrence, and complication rates were 6.9% versus 29.9%, 2.1% versus 10.9%, and 6.4% versus 6.6%, respectively). Surgery remained superior in terms of initial occlusion rate and recurrence and equivalent with respect to complication rates. At the same time, we observed an improvement, in comparison with earlier meta-analysis, of failed initial obliteration [88] and recurrence rates [89,91] for embolism, a fact that can be attributed to experience accumulation and better embolic agent technologies during the last 10 years, but still not enough to render embolism more successful than surgery. No report of better clinical outcomes attributed to one of the two modalities was found. Bearing upon one of our cases (case 1), we intend to emphasize, with respect to surgical technique, the need for thorough preoperative DSA, as - though infrequently - feeders may be multiple [36,92] and the necessity of obliterating the shunt intradurally and directly, as the disconnection of the vein with the intradural reflux may not suffice to prevent a recurrence [4,6]. We also assert the need for close postoperative follow-up, as deterioration is possible, either due to intraoperative technical difficulties or as an anticipated complication of the disease’s course.

Progression during the delay period and asymptomatic patients

Thirteen [12,18,28,31,40,43,44,46,58,67,69,74,82] studies discussed the progression of the disease during the delay-to-diagnosis period or after-a-failed-intervention period, and proper management of extreme cases of asymptomatic or late-stage patients. In view of the former, sDAVFs are already notorious for their creeping progression, even when directly compared to other spinal cord ischemia causes [40]. Eight [12,18,28,43,44,46,69,82] studies examined disease progression during the delay period until a definite diagnosis or until definite treatment. Five [12,31,43,69,82] of them showed significant disease progression, in contrast to three that did not [18,44,46]. With respect to the second question of optimal management in the asymptomatic or minorly symptomatic patient, four studies [18,28,44,82] argue in favor of a conservative “wait and see” strategy, all of them referring to CCJ and cervical sDAVFs. Conversely, two studies report on the late-stage patient, causing controversy. Although one showed poor potential for an ambulance [74], the other [58] suggests that aggressive treatment should be attempted as expected McCormick score (MCS) improvement of 1 implies that the patient may still be partially dependent, rather than plegic.

What to do with the asymptomatic patient?

The natural history of sDAVFs creates a debate about the appropriate time of intervention. Aminoff and Logue showed that half of the patients will become severely disabled (non-ambulatory) within 3 years [93]. Moreover, it has been shown that even a patient with a severe preoperative status, when presented acutely, can benefit from early intervention [94]. Therefore, it is unequivocal that symptomatic patients should be treated. On the other hand, it has been suggested that asymptomatic patients should be closely followed (wait-and-see strategy), given that only 20% are expected to become symptomatic [44]. Though thoracolumbar sDAVFs can be managed more aggressively - that is, an intervention may be appropriate even in asymptomatic patients, and the indication depends on center and surgeon experience [44]- management of cervical and CCJ sDAVFs should be more conservative, with regular and frequent follow-up of the patient before advancing to treatment [18], in favor of surgery, given the more complex angioarchitecture [55]. Given that in our review, five out of eight studies showed disease progression during the wait-and-see period and that the prognostic value of delayed diagnosis and preoperative status is generally accepted [24], the patient should be informed of the probable natural course of the disease, and the decision should be made jointly by both the surgeon and the patient and in case of conservative management, the close follow up should be accepted by both.

Limitations

The present systematic review of the literature offers a simple pooled statistical analysis to accompany the updated knowledge. The studies contributed to simple statistics and showed notable heterogeneity. Heterogeneity was expressed in various ways through population differences between (different scales of clinical status, scattered reports, and fragmented variables) and within (many case series reported mixed data on dural and extradural shunts) studies. In addition, there is a heterogeneous grouping of embolism agents, such as n-Butyl Cyanoacrylate (nBCA), Onyx^TM^ (Medtronic, Minneapolis, USA), or polyvinyl alcohol, which may vary in multiple aspects (for example, OnyxTM has lower recurrence rates than nBCA [91]), as well as operative techniques (some studies used intraoperative imaging, whereas others did not).

We found similar complication rates between the two modalities, though numbers were slightly higher. This can either be explained by more aggressive management or could just be attributed to the heterogeneity of the studies with respect to their main focus topic; some studies focused on imaging characteristics, rather than reporting complications. It is reasonable to assume that a study focused on a subject different than intervention-related events could be biased towards not mentioning a low or zero complication rate, therefore, in grouping the results, we miss those lower rates and the aggregate rate increases. Finally, many studies did not elaborate on indications and decided first-line intervention based on institute common practice and experience.

## Conclusions

SDAVF is a rare entity, usually encountered in middle-aged males with delayed definite diagnosis. A diagnosis of transverse or ascending myelitis referred to the neurosurgeon should always raise suspicion. No prognostic factor remains unchallenged, though preoperative clinical status is the most commonly acknowledged and can guide the management. Lately, new MRI sequences, such as TRICKS and SPACE have reached localization sensitivity equivalent to DSA. The two intervention modalities, operative and endovascular, are equivalent with respect to clinical outcome and complication rates. However, surgery has a better definitive treatment rate, regarding initial fistula occlusion and recurrence. Embolism can be offered as first-line treatment in patients undergoing DSA, but this depends on center expertise and patient profile, as contraindications can preclude patients from embolism. Conservative management can be offered only in asymptomatic patients and only if patient education is guaranteed with a strict follow-up agreed upon by both the clinician and the patient.
